# Gamma-Secretase Represents a Therapeutic Target for the Treatment of Invasive Glioma Mediated by the p75 Neurotrophin Receptor

**DOI:** 10.1371/journal.pbio.0060289

**Published:** 2008-11-25

**Authors:** LiMei Wang, Jennifer J Rahn, XueQing Lun, Beichen Sun, John J. P Kelly, Samuel Weiss, Stephen M Robbins, Peter A Forsyth, Donna L Senger

**Affiliations:** 1 Department of Oncology, University of Calgary, and Tom Baker Cancer Centre, Calgary, Canada; 2 Department of Biochemistry and Molecular Biology, University of Calgary, Calgary, Canada; 3 Clark H. Smith Brain Tumor Center and Southern Alberta Cancer Research Institute, Calgary, Canada; 4 Department of Cell Biology and Anatomy, University of Calgary, Calgary, Canada; 5 Hotchkiss Brain Institute, Calgary, Canada; 6 Department of Clinical Neurosciences, University of Calgary, Calgary, Canada; Fred Hutchinson Cancer Research Center, United States of America

## Abstract

The multifunctional signaling protein p75 neurotrophin receptor (p75^NTR^) is a central regulator and major contributor to the highly invasive nature of malignant gliomas. Here, we show that neurotrophin-dependent regulated intramembrane proteolysis (RIP) of p75^NTR^ is required for p75^NTR^-mediated glioma invasion, and identify a previously unnamed process for targeted glioma therapy. Expression of cleavage-resistant chimeras of p75^NTR^ or treatment of animals bearing p75^NTR^-positive intracranial tumors with clinically applicable γ-secretase inhibitors resulted in dramatically decreased glioma invasion and prolonged survival. Importantly, proteolytic processing of p75^NTR^ was observed in p75^NTR^-positive patient tumor specimens and brain tumor initiating cells. This work highlights the importance of p75^NTR^ as a therapeutic target, suggesting that γ-secretase inhibitors may have direct clinical application for the treatment of malignant glioma.

## Author Summary

Despite technical advances, clinical prognosis of patients with malignant glioma, with an average survival of less than one year, has not changed. The highly invasive nature of these tumors, together with the recently identified brain tumor-initiating cells, provide disease reservoirs that render these tumors incurable by conventional therapies. Here, we present the first evidence to our knowledge that regulated intramembrane proteolysis of the neurotrophin receptor p75^NTR^ is a critical regulator of glioma invasion. Inhibition of this process by clinically relevant γ-secretase inhibitors dramatically impairs the highly invasive nature of genetically distinct glioblastomas and brain tumor-initiating cells and prolongs survival. These data highlight regulated intramembrane proteolysis as a therapeutic target of malignant glioma and implicate the application of γ-secretase inhibitors in the treatment of these devastating tumors.

## Abstract 3

Gamma-secretase inhibitors in clinical trials for patients with Alzheimer disease can be used to block the highly invasive behavior of malignant glioma and prolong survival.

## Introduction

Human malignant glioma (MG) is one of the most common primary central nervous system tumors in adults. These tumors are diffuse, highly invasive, with dismal prognosis, and long-term survivors are rare [1,2]. MG extend tendrils of tumor several centimeters away from the main tumor mass. These, as well as the recently identified brain tumor-derived stem-like cells [3–6], herein called brain tumor-initiating cells (BTICs), act as “disease reservoirs,” rendering these tumors refractory to available treatments such as surgery or radiotherapy [7,8]. The highly invasive nature of these tumors is the result of genotypic and phenotypic changes that result in the activation of a number of coordinate cellular programs, including those necessary for migration (e.g., motility) and invasion (e.g., extracellular matrix [ECM] degradation) [9] and changes in pathway signaling that impart resistance to conventional treatments by reducing proliferation and increasing resistance to apoptosis [8,10,11]. A detailed understanding of the mechanisms underlying this invasive behavior is essential for the development of effective therapies.

Several genes, including those that encode uPA/uPAR, ephrinB3/EphB2, matrix metalloproteinases (MMPs), a disintegrin and metalloproteases (ADAMs), cathepsins, and integrins, have previously been implicated in glioma invasion [12]. More recently, gene expression profiling identified several subclasses of gliomas that separate tumors into good and poor prognosis groups of which diffuse infiltrative gliomas are divided into four such subclasses [13]. One of these four subclasses, designated hierarchical cluster 2B (HC2B), was found to include several genes with specific roles in cell migration and invasion, and membership in this group was found to strongly correlate with poor patient survival. Our understanding of the proteins that initiate, and the pathways that regulate, glioma invasion is continually expanding, such as the recent discovery that CD95 via the activation of the PI3K/Akt/glycogen synthetase kinase (GSK3β) pathway regulates glioma invasion [14]. However, despite recent advances and efforts to target these specific molecules or pathways, no clinically relevant agents have been identified as yet. Using a discovery-based approach and a series of functional, biochemical, and clinical studies, we have identified the p75 neurotrophin receptor (p75^NTR^) as a critical regulator of glioma invasion [15]. We found that p75^NTR^, through a neurotrophin-dependent mechanism, dramatically enhanced migration and invasion of genetically distinct glioma and that robust expression of p75^NTR^ was detected in the highly invasive tumor cell population from p75^NTR^-positive glioblastoma patient specimens. In this current study, we investigated the mechanism by which p75^NTR^ imparts this highly invasive behavior to malignant glioma, and assessed the use of a clinically applicable agent in abrogating this invasive behavior.

p75^NTR^ elicits a large array of diverse biological responses that are regulated by a complex layer of mechanisms. These intricate layers of control have been proposed to explain the variety of cellular effects triggered by p75^NTR^ activation. Key p75^NTR^ signaling pathways already identified include Ras homolog gene family, member A (RhoA), Jun N-terminal kinase (JNK), mitogen-activated protein kinase (MAPK), and nuclear factor κ B (NFkB) [16]. These pathways are believed to be activated by upstream proteins that directly associate with various regions of the p75^NTR^ intracellular domain (ICD). These proteins include guanine nucleotide dissociation inhibitor (RhoGDI), ribosome-inactivating protein-2 (RIP-2), and p75^NTR^-associated cell death executor (NADE) [17–20], which associate with a region referred to as the “death domain”; Schwann cell factor-1 (SC-1); neurotrophin receptor-interacting MAGE homolog (NRAGE); tumor necrosis factor (TNF) receptor-associated factor (TRAF), and neurotrophin receptor interacting factor (NRIF) [21–23], which associate with the juxtamembrane region of p75^NTR^; and a PDZ-containing protein Fas-associated phosphatease-1 (FAP-1), which associates with the C-terminal Ser-Pro-Val (SPV) [24]. What proteins or biological process are activated by p75^NTR^, however, is highly cell context specific. In addition to associating with other signaling molecules, p75^NTR^, similar to amyloid precursor protein (APP) and Notch, has been shown to undergo regulated α-secretase and γ-secretase cleavage, referred to as regulated intramembrane proteolysis (RIP). Cleavage of several type-1 transmembrane receptors has been implicated and shown to be necessary in eliciting some downstream biological responses [25–28]. α-Secretase cleavage of full-length p75^NTR^ by a sheddase liberates the extracellular domain (ECD), leaving an unstable membrane-bound C-terminal fragment (CTF) that is cleaved by the γ-secretase complex to release an ICD with potential signaling capability [26,29]. Here, we show for the first time to our knowledge that regulated intramembrane proteolysis of p75^NTR^ is a requirement for the highly invasive behavior of p75^NTR^-positive malignant glioma, and designate RIP as a clinical target for the treatment of invasive malignant glioma.

## Results

### p75^NTR^ Proteolytic Processing Occurs in Patient Specimens Endogenously Expressing p75^NTR^

**Figure 1:**
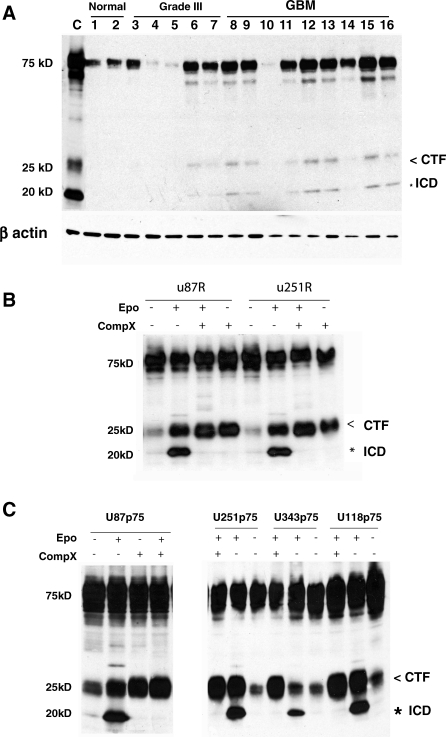
p75^NTR^ Is Proteolytically Processed in Patient Specimens and Invasive Glioma Cells (A) Western blot analysis detected p75^NTR^-positive fragments migrating at 24 and 19 kDa in patient specimens expressing p75^NTR^ (eight of nine GBMs, two of five Grade III glioma) but was undetectable in tissue from normal human brain. U87 cells transfected with p75^NTR^ and grown in the presence of the proteasome inhibitor epoxomicin (1 μM) were used as a positive control (C). Western blots probed for beta-actin were used as a loading control. Molecular weight markers are indicated on the left. (B) The highly invasive glioma cell lines U87R and U251R isolated by serial in vivo selection were treated with the proteasome inhibitor epoxomicin (Epo, 1 μM) and/or the γ-secretase inhibitor, Compound X (CompX, 2 μM) for 4 h. Western blots for p75^NTR^ were probed with an antibody specific to the cytoplasmic domain of p75^NTR^ which detects full-length (75 kDa), CTF (indicated by the less than symbol [<]; 25 kDa), and ICD (indicated by the asterisk [*]; 19 kDa) peptides. The ICD is derived from the cleavage of the CTF, which is shown to visibly accumulate in the presence of a γ-secretase inhibitor. (C) p75^NTR^ proteolytic processing is a global event in human glioma cells. pcDNA3.1 encoding human p75^NTR^ or the empty pcDNA3.1 vector were stably transfected into U87, U251, U118, and U343. Cells expressing p75^NTR^ (U87p75, U251p75, U118p75, and U343p75) were treated as described above. Western blot analysis showed that all glioma cell lines tested cleaved the full-length p75^NTR^ to generate first the 24-kDa CTF and then the 19-kDa ICD.

In a previous study, our laboratory identified p75^NTR^ as a potent mediator of invasion in human glioma using a novel invasive glioma mouse model generated by serial in vivo selection [15]. In that study, we found that p75^NTR^ was expressed in 22% mid-grade astrocytomas (two of nine) and 85% of glioblastoma multiforme (GBM) specimens (17 of 20), and that the p75^NTR^-positive glioma cells in the patient tumor cell population were more migratory than the p75^NTR^-negative glioma cells. Here, we investigate the mechanism underlying this p75^NTR^-induced invasion. In neurons, p75^NTR^ is a substrate for sequential α- and γ-secretase–mediated intramembrane proteolysis generating 24 kDa CTF and 19 kDa ICD fragments, respectively, and the generation of these fragments are required for some of its biological functions [26,28,30–34]. We therefore sought to determine whether intramembrane proteolysis of p75^NTR^ occurred in malignant glioma patient specimens. To do this, we assessed whether the generation of the 24-kDa CTF and the 19-kDa ICD occurred in a panel of surgically resected human glioma specimens and normal human brain. Tumor and normal tissue taken at the time of surgery were immediately snap frozen in liquid nitrogen and stored at −80 °C. Frozen tumor tissue was digested in lysis buffer and analyzed by western blots using a p75^NTR^ cytoplasmic-specific antibody that not only detected the full-length p75^NTR^ protein, but also detected p75^NTR^-positive fragments migrating at 24 and 19 kDa, respectively, in the p75^NTR^-positive specimens (eight of nine GBMs and two of five Grade III glioma) (Figure 1A). Hence, p75^NTR^ processing occurs in human glioma tumors, and this suggested the possibility that p75^NTR^ processing is required for glioma invasion.

### p75^NTR^ Is Proteolytically Processed in Invasive Glioma Cells

To address the possible role(s) of p75^NTR^ proteolytic processing in glioma cells, we assessed whether the appearance of the p75^NTR^-positive fragments at 24 and 19 kDa was the result of proteolytic processing of the full-length p75^NTR^ in invading glioma cells. We have previously established the highly invasive glioma cell lines U87R and U251R for which p75^NTR^ accounts for their invasive behavior [15]. The U87R and U251R invasive glioma cells were grown in the absence or presence of the proteasome inhibitor epoxomicin, a compound used to inhibit rapid degradation of proteins often associated with RIP-mediated proteins [26,35]. Western blot analysis showed that in addition to the 75-kDa full-length p75^NTR^ protein, 24-kDa and 19-kDa fragments (Figure 1B, lane 1 and 5) were present and stabilized in the presence of 1 μM epoxomicin (Figure 1B, lane 2 and 6). These results are in agreement with the model that the full-length p75^NTR^ protein is cleaved, releasing the ECD, CTF, and intracellular fragments. Next, we verified that the appearance of the 24-kDa and 19-kDa fragments was the result of sequential cleavage of p75^NTR^ by an α-secretase and then a γ-secretase. First, we determined whether treatment of p75^NTR^ glioma cells (U87p75^NTR^) using the TNF-α protease inhibitor (TAPI)-2, known to inhibit metalloproteases and ADAMs such as tumor necrosis factor-α converting enzyme (TACE) [36–38] and previously shown to inhibit the proteolytic processing of p75^NTR^ in neurons [26,32,33,39], could inhibit p75^NTR^ processing in glioma cells. TAPI-2 inhibited the proteolytic processing of p75^NTR^ as indicated by the lack of CTF and ICD, and abrogated p75^NTR^-mediated invasion (Figure S1). Since TAPI-2 has broad specificity, and glioblastomas are known to produce high levels of many proteases, including members of the MMP, ADAM, and ADAMTS families, leaving the exact identity of the α-secretase unclear, we focused our efforts on the second cleavage event. To determine whether the generation of the 19-kDa fragment was the result of cleavage of p75^NTR^ by a γ-secretase, U87R and U251R cells were treated with 2 μM Compound X (Calbiochem), a specific inhibitor of γ-secretase, for 4 h in the absence or presence of epoxomicin. Western blot analysis of p75^NTR^ revealed that in the presence of the γ-secretase inhibitor, an accumulation of the 24-kDa fragment occurred without subsequent cleavage to the 19-kDa ICD, consistent with the release of the ICD of p75^NTR^ by γ-secretase (Figure 1B, lanes 3, 4, 7, and 8). The role of processing of p75^NTR^ was not limited to a single glioma cell line and was a general mechanism observed in glioma cells established from genetically distinct individuals (U87p75, U251p75, U343p75, and U118p75).We found that in all p75^NTR^-positive glioma cell lines, full-length p75^NTR^ was cleaved to generate two fragments of 19 and 24 kDa: ICD and CTF, respectively (Figure 1C). These results demonstrate that regulated intramembrane proteolysis of p75^NTR^ is a global event occurring in highly invasive p75^NTR^-positive human glioma cells.

### γ-Secretase Inhibition Significantly Abrogated p75^NTR^-Induced Glioma Migration and Invasion In Vitro

**Figure 2:**
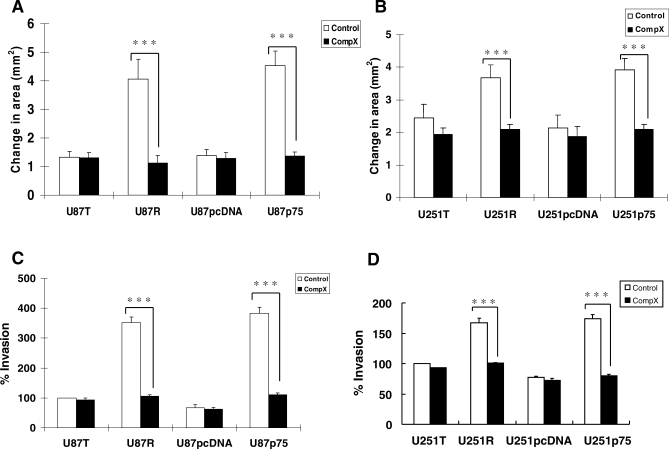
γ-Secretase Inhibitor Significantly Inhibited p75^NTR^-Induced Migration and Invasion in Glioma Cells (A and B) Monolayer circular migration assays of U87R and U251R cells, which endogenously express p75^NTR^, and U87p75 and U251p75 cells, which ectopically express p75^NTR^, were performed. Briefly, cells were seeded in monolayer wells through a cell sedimentation manifold. p75^NTR^-negative U87T, U87pcDNA, U251T, and U251pcDNA cells were used for comparison. Once the sedimentation manifolds were removed, cells were given complete medium containing 2 μM Compound X (CompX). Digital images of the cells were taken before migration at 0 h and then again at 72 h. Best-fit circles were drawn around the area covered by the cells at the 0 h and 72 h time points, and the actual cell area was determined using Axiovision 4.2 imaging software. Quantitative migration scores were calculated as the increase in the area covered by the cells beyond the initial area of the cells. γ-Secretase inhibitor Compound X significantly inhibited p75^NTR^-induced migration. Values shown are the mean ± s.e.m. from three independent experiments; triple asterisks (∗∗∗) indicate *p* < 0.001 as compared to control (one-way analysis of variance [ANOVA] with the Neuman-Keuls post-test). (C and D) 3D-collagen invasion assays of U87R, U87p75, U251R, and U251p75 cells were performed by mixing cells with 3D-collagen matrix (collagen, fibronectin, and laminin) and then seeding them into 8.0-μm pore size transwell chambers in the presence or absence of 2 μM Compound X for 6 h. Cells were fixed and stained, and invasive cells were counted. U87T, U87pcDNA, U251T, and U251pcDNA cells were used for comparison. Inhibition of γ-secretase by Compound X significantly inhibited p75^NTR^-induced glioma invasion. Values shown are the mean ± s.e.m. from three independent experiments; triple asterisks (***) indicate *p* < 0.001 as compared to control (one-way ANOVA with the Neuman-Keuls post-test).

**Figure 3:**
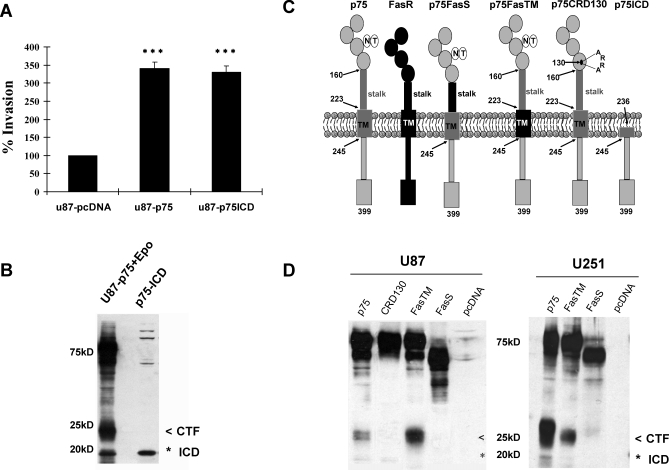
Expression and Proteolytic Processing of p75^NTR^ Chimeric Alleles (A) p75^NTR^ ICD is sufficient to induce invasion of U87 glioma cells in vitro. 3D-collagen invasion assays of U87p75ICD cells were performed by mixing cells with 3D-collagen matrix (collagen, fibronectin, and laminin) and then seeding them into 8.0-μm pore size transwell chamber for 6 h. Cells were fixed and stained, and invasive cells were counted. U87pcDNA and U87p75 cells were used for comparison. Values shown are the mean ± s.e.m. from three independent experiments; triple asterisks (***) indicate *p* < 0.001 as compared to U87pcDNA (one-way ANOVA with the Neuman-Keuls post-test). (B) Western blot analysis detected a p75^NTR^-positive fragment migrating at 19 KDa in U87 cells expressing the p75-ICD construct. U87p75^NTR^ glioma cells treated with the proteasome inhibitor epoxomicin (Epo, 2 μM) were used as a positive control (U87-p75+Epo; the less than symbol [<] indicates CTF; the asterisk [*] indicates ICD). (C) Schematic diagram of the p75^NTR^ constructs. Chimeric proteins were created by replacing either the transmembrane (TM) or the extracellular stalk (S) domain of p75^NTR^ with equivalent domains from the Fas receptor. The proteins were designated *p75FasTM* (FasTM) and *p75FasS* (FasS), respectively. Other p75^NTR^ constructs were generated, including the neurotrophin-binding mutant in which the ligand-binding site was mutated by inserting four amino acids after amino acid residue 130, designated *p75CRD130* (*CRD130*), and the p75^NTR^ intracellular domain construct consisting of amino acids 238 to 399 (*p75-ICD*). (D) Western blot analysis using a polyclonal p75^NTR^ antibody to the intracellular domain of p75^NTR^ confirmed the proper expression and processing of all p75^NTR^ constructs (*p75FL*, *p75FasTM*, *p75FasS*, or *p75CRD130*) in both U87 (left panel) and U251 (right panel) human glioma cell lines (the less than symbol [<] indicates CTF; the asterisk [*] indicates ICD). To examine comparable levels of chimeric proteins, lysates were adjusted accordingly.

In neurons, ectodomain shedding of p75^NTR^ by α-secretase and then γ-secretase cleavage to generate an ICD fragment can result in the activation of downstream events [26–28,30–34]. To test whether the processing of p75^NTR^ resulting in the release of the ICD fragment has a functional role in glioma invasion, we analyzed in vitro migration and invasion of U87R, U251R, U87p75^NTR^, and U251p75^NTR^ glioma cell lines using circular monolayer migration assays (Figure 2A and 2B) and 3D-collagen invasion assays (Figure 2C and 2D) in the absence and presence of the γ-secretase inhibitor, Compound X. p75^NTR^-mediated glioma migration and invasion were significantly inhibited in the presence of Compound X. In contrast, when the proteasome inhibitor epoxomicin was used to stabilize p75^NTR^-ICD, a significant increase in migration and invasion was seen (unpublished data), consistent with increased invasion observed when a cDNA construct mimicking the ICD fragment was ectopically expressed in U87 glioma cells (Figure 3Aand 3B). To determine whether γ-secretase inhibition was confined to glioma invasion or had effects on other biological processes, we assessed the effect of γ-secretase inhibition on survival and proliferation of p75^NTR^-positive glioma cells. No significant change was observed on either survival or proliferation in vitro (Figure S2).

### Biochemical Characterization of U87 and U251 Glioma Cells Expressing p75^NTR^ Wild-Type or Cleavage-Resistant Chimeric p75^NTR^ Alleles

It is well known that γ-secretase has many substrates [40,41]. To directly test the role of p75^NTR^ processing in glioma invasion, we constructed cleavage-resistant chimeric proteins of p75^NTR^ by replacing either the transmembrane (*p75FasTM*) or the extracellular stalk domain of p75^NTR^ (*p75FasS*) with equivalent domains from the Fas receptor [39] (Figure 3C). Both p75^NTR^ and Fas receptors are members of the TNF receptor superfamily, and although they each contain similar domains, Fas, unlike p75^NTR^, does not undergo RIP. Since ectopic expression of p75^NTR^ in the human glioma cells lines U87 and U251 was sufficient to mediate glioma invasion [15], these cell lines were used as a model system to assess the p75^NTR^ chimeric mutants. U87 and U251 were therefore stably transfected with the cleavage-resistant p75^NTR^ constructs (*p75FasS* and *p75FasTM*). To ensure proper function of all p75^NTR^ protein constructs, we assessed their location, topography, and ability to bind neurotrophin in both U87 and U251 glioma cell lines. Receptor orientation and localization at the plasma membrane was confirmed by flow cytometric analysis using a monoclonal antibody specific to the ECD domain of p75^NTR^ (Figure S3A). As expected, all p75^NTR^ constructs were expressed at the plasma membrane with the correct topography. Next, we assessed whether the chimeric constructs could still bind neurotrophin. Previously, we demonstrated that in the absence of p75^NTR^, glioma cells secrete high levels of brain-derived neurotrophic factor (BDNF) protein into the culture medium in vitro. When these same cells express p75^NTR^, the majority of the BDNF is found to be cell associated, presumably bound to p75^NTR^ [15]. To confirm that the p75^NTR^ cleavage-resistant chimeric forms retained the ability to bind neurotrophin, ELISA assays were performed to detect BDNF expression in the conditioned medium and total cell lysates of U87 and U251 cells expressing *p75FL*, *p75FasTM*, *p75FasS*, and *p75CRD130* (Figure S3B). *p75CRD130* is a neurotrophin-binding mutant created by inserting four amino acids after amino acid residue 130 [15,42–47]. Expression of the chimeric p75^NTR^ proteins (*p75FasTM* and *p75FasS*), just like the p75 wild type, resulted in a shift in BDNF localization from the conditioned medium to the cell lysate. This was in contrast to the cells expressing the neurotrophin-binding mutant (*p75CRD130*) or the empty vector (pcDNA) where the bulk of BDNF was detected in the culture medium. These data demonstrate that p75^ NTR^ cleavage-resistant chimeric constructs *p75FasTM* and *p75FasS* retained their ability to bind neurotrophin (Figure S3C). Once we confirmed the correct expression and binding of the various p75^NTR^ constructs, western blots using a p75^NTR^ cytoplasmic domain-specific antibody were performed to evaluate proteolytic processing of the various p75^NTR^ receptors (Figure 3D). In cells expressing *p75FasS*, only the full-length protein was detected, consistent with inhibition of the α-secretase cleavage, whereas the full-length 75 kDa and the 24-kDa fragment were detected in cells expressing the *p75FasTM* construct corresponding to the ectodomain shedding of p75^NTR^ by α-secretase but with inhibition of the γ-secretase cleavage. Moreover, in the presence of epoxomicin, no additional p75^NTR^ fragments were observed (Figure S4). These results demonstrate the cleavage-resistant chimeric p75^NTR^ alleles were expressed with correct biochemical characteristics in U87 and U251 glioma cells. In addition, and consistent with the hypothesis that proteolytic processing of p75^NTR^ is required for glioma invasion, only the full-length 75 kDa band was detected in lysates from U87 cells expressing *p75CRD130*, a p75^NTR^ construct that was unable to induce glioma invasion [15].

### Cleavage-Resistant Chimeric Forms of p75^NTR^ Do Not Induce Glioma Migration and Invasion In Vitro or In Vivo

**Figure 4:**
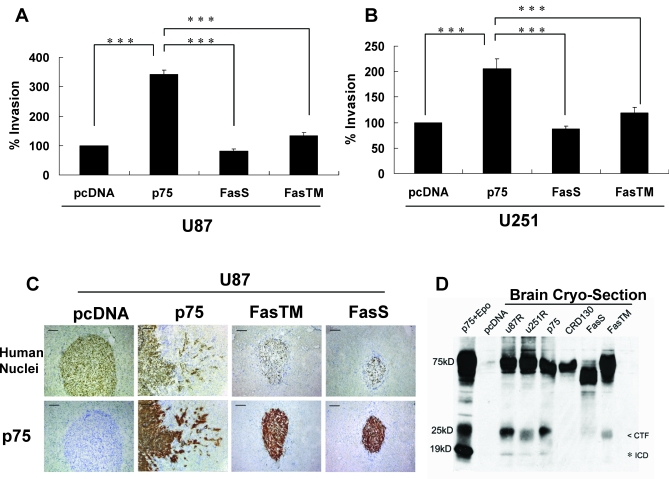
Cleavage-Resistant Chimeric p75^NTR^ Proteins Do Not Induce Migration and Invasion In Vitro or In Vivo (A and B) The invasive ability of U87 (A) and U251 (B) stably transfected with p75^NTR^ or the p75^NTR^ cleavage-resistant chimeric constructs (*p75FasS* and *p75FasTM*) were assessed using 3D-invasion assays. Expression of p75^NTR^ significantly increased invasion in the genetically distinct glioma cell lines U87 and U251, whereas neither *p75FasS* nor *p75FasTM* stimulated glioma invasion as compared to the p75^NTR^-negative control cells (pcDNA). Values shown are the mean ± s.e.m. from three independent experiments; (the less than symbol [<] indicates CTF; the asterisk [*] indicates ICD; triple asterisks [∗∗∗] indicate *p* < 0.001 vs. p75^NTR^ wild type, one-way ANOVA with Neuman-Keuls post-test). (C) U87 cells stably expressing p75^NTR^ or the cleavage-resistant *p75FasS* and *p75FasTM* were implanted into the brains of SCID mice and allowed to grow for 28 d. The mice were sacrificed, and frozen brain sections were stained with antibodies against human nuclei (top row, brown) and human p75^NTR^ (bottom row, brown). Sections were counterstained with toluidine blue (blue). Scale bars in (C) represent 100 μm. Implantation of U87 glioma cells stably transfected with the empty pcDNA vector or p75^NTR^ cleavage-resistant chimeras (*p75FasTM* and *p75FasS*), led to the formation of well-circumscribed tumors. In marked contrast, animals implanted with U87 glioma cells ectopically expressing p75^NTR^ (U87p75) developed tumors with highly infiltrative edges. Similar results were seen in three independent experiments with three animals in each group. (D) p75^NTR^ cleavage-resistant chimeras do not generate ICD in vivo. Brain cryosections from SCID mice implanted with U87 glioma cells expressing p75^NTR^, *p75FasTM*, or *p75FasS* were lysed in 2× loading buffer. Proteins were resolved on 10% SDS-PAGE gels and probed with a p75 cytoplasmic-specific antibody. U87R, U251R, and U87p75, which express high levels of p75^NTR^ and exhibit increased invasive activity, process full-length p75^NTR^ to generate both CTF and ICD in vivo. In contrast, and consistent with the in vitro data, the p75^NTR^ ICD peptide was not detected in tumors from animals expressing *p75FasS* or *p75FasTM*. Cell lysates from U87 cells expressing p75^NTR^ and grown in the presence of epoxomicin (p75^NTR^+Epo) or expressing pcDNA (pcDNA) were used as positive and negative controls, respectively.

Since we have shown that neurotrophin binding is required for p75^NTR^-mediated glioma invasion [15], and the neurotrophin-binding mutant *p75CRD130* does not undergo RIP, it would appear that RIP of p75^NTR^ is required for glioma invasion. To determine whether this is in fact true, U87 and U251 cells expressing the p75^NTR^ cleavage-resistant constructs were assessed for their invasive ability using 3D-collegen invasion assays. We found that expression of cleavage-resistant forms of p75^NTR^ (*p75FasS*, *p75FasTM*, and *p75CRD130*), which prevented receptor proteolysis, blocked p75^NTR^-mediated glioma invasion (Figure 4A and 4B), providing evidence to support a role for γ-secretase–dependent release of p75^NTR^ ICD in mediating glioma invasion.

To determine whether p75^NTR^ processing was required for glioma invasion in vivo, U87 glioma cell lines ectopically expressing *p75FasS* and *p75FasTM* were implanted into the brains of immunocompromised (SCID) mice. U87 glioma cells expressing full-length p75^NTR^ (U87p75) or control vector (U87pcDNA) were used for comparison. Twenty-eight days after implantation, the mice were sacrificed, and frozen brain sections were stained with antibodies against human nuclei, to visualize all glioma cells (Figure 4C, upper panel) or with anti-human p75^NTR^ (Figure 4C, bottom panel). Implantation of U87 glioma cells stably transfected with the control pcDNA vector led to the formation of well-circumscribed tumors, while U87 glioma cells ectopically expressing p75^NTR^ formed tumors with highly infiltrative edges. In sharp contrast to the p75^NTR^-expressing U87 tumors, tumors expressing either *p75FasTM* or *p75FasS* formed well-circumscribed tumors similar to the p75^NTR^-negative tumors (U87pcDNA). Comparable results were seen in three independent experiments. In conjunction with the in vitro data, these data suggest that proteolytic processing of p75^NTR^ is required for glioma invasion in vivo (Figure 4C).

It is well known that the microenvironment of tumors can change the biochemical characterization and function of cells. We have demonstrated in vitro that glioma cells expressing p75^NTR^ undergo proteolytic processing to generate first the 24-kDa CTF and then the 19-kDa ICD. To provide evidence that RIP of p75^NTR^ occurs in vivo, 7–9-μm cryosections from mice implanted for 3–4 wk with in vivo–selected U87R and U251R, or ectopically expressing p75^NTR^, *p75FasS*, *p75FasTM*, and pcDNA, were assessed for p75^NTR^ processing by western blot. The 24- and 19-kDa fragments were found in the highly invasive glioma cells, U87R, U251R, and U87p75^NTR^. In contrast, neither the 24-kDa nor the 19-kDa fragment was seen in cells expressing *p75FasS*, and as expected, only the 24-kDa fragment was detected in cells expressing the *p75FasTM*, consistent with their in vitro characterization (Figure 4D). These data further support a role for RIP of p75^NTR^ in glioma invasion.

### p75^NTR^ Proteolytic Processing Occurs in BTICs from Patient Specimens That Endogenously Express p75^NTR^

**Figure 5:**
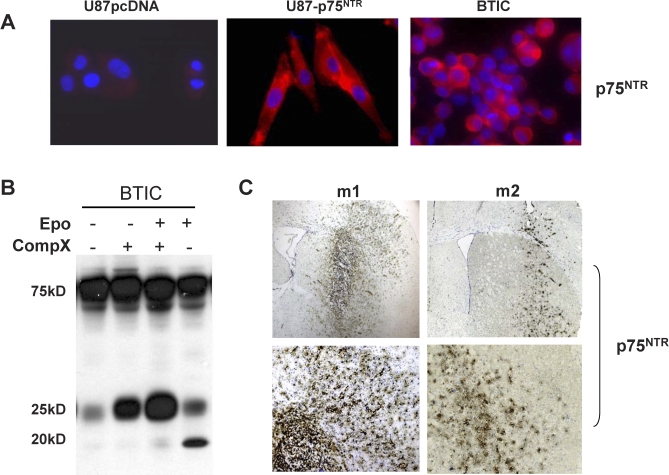
Regulated Intramembrane Proteolysis Occurs in Brain Tumor-Derived Stem-Like Cells (BTICs) from Glioma Patient Specimens (A and B) Primary cultures from human glioma patient tumor grown under brain tumor stem cell-promoting conditions (BTIC) express high levels of p75^NTR^ (red) in vitro as detected by immunocytochemistry (A) and western blot (B). U87 cells stably expressing pcDNA and p75^NTR^ were used as negative and positive controls, respectively. Cultures were counterstained with DAPI (blue) to visualize the cell nucleus. (B) p75^NTR^ expressed on the BTICs undergoes RIP. BTICs were grown in the absence and presence of 2 μM γ-secretase and/or 1 μM epoxomicin. Western blot analysis detected full-length p75^NTR^, CTF, and ICD with the accumulation of the 24-kDa CTF in the presence of the γ-secretase inhibitor (CompX). (C) Brain tumor-initiating cells (BTIC) express p75^NTR^ in vivo. BTIC cells were implanted into the brains of SCID mice and allowed to establish for 4–8 wk. Animals were sacrificed, and frozen sections were stained with anti-p75^NTR^ (brown). Sections were counterstained with toluidine blue (blue). Tumors established from BTICs express high levels of p75^NTR^ and form highly infiltrative tumors. M1 and M2 are individual mice.

Our data demonstrated that 85% of GBM specimens (17/20) express p75^NTR^, that the p75^NTR^-positive glioma cells in the original patient tumor cell population were more migratory [15], and that 24- and 19-kDa p75^NTR^-positive fragments are present in p75^NTR^-positive primary Grade III and GBM patient specimens (Figure 1). We therefore wanted to determine whether the appearance of these fragments in the malignant glioma patient specimens was the result of intramembrane proteolysis of p75^NTR^. To do this, we established primary cultures from human glioma patient tumors. The recent discovery that human stem cell-like tumor cells, termed BTICs, retain characteristics that closely recapitulate the original patient tumor [3–6,48,49] prompted us to establish the primary patient tumor cultures under neural stem cell–promoting conditions. BTICs share characteristics with neural stem cells (NSCs) such as continuous self-renewal, extensive brain parenchymal migration and infiltration, and potential for full or partial differentiation, properties not found in established glioma cell lines [50,51]. Operative samples of human GBM were obtained at the time of surgery, and brain tumor sphere cultures were established in NS-A basal medium plus epidermal growth factor (EGF) and basic fibroblast growth factor (bFGF) (EF medium). Immunocytochemical analysis of BTICs established from five glioma patients expressed the early neural cell progenitor proteins Nestin, Musashi, hSox2, and CD133 (J. J. P. Kelly, S. Weiss, P. A. Forsyth, and D. L. Senger; unpublished data). In addition, four out of five glioma tumor progenitor cells in vitro expressed high levels of p75^NTR^ as detected by immunocytochemistry (Figure 5A) and western blot (Figure 5B). To determine whether p75^NTR^ expressed on the BTICs undergoes RIP, BTICs were grown in the absence and presence of γ-secretase and/or 2 μM epoxomicin. Similar to the glioma cell lines, full-length p75^NTR^, CTF, and ICD were detected, and the 19-kDa ICD fragment was dependent on γ-secretase cleavage (Figure 5B). We next determined whether the BTICs retained their expression of p75^NTR^ in vivo: BTIC cells were implanted into the brains of SCID mice and allowed to establish for 4–8 wk. Animals were sacrificed, and frozen sections were stained with either an anti-human nuclear antibody (to identify the human BTICs) or anti-p75^NTR^ (Figure 5C). All tumors established from BTICs cells showed highly infiltrative tumors, and consistent with the in vitro data, four out of five tumors showed high expression of p75^NTR^ in vivo.

### γ-Secretase Inhibitor Significantly Abrogated p75^NTR^-Induced Migration and Invasion In Vivo

**Figure 6:**
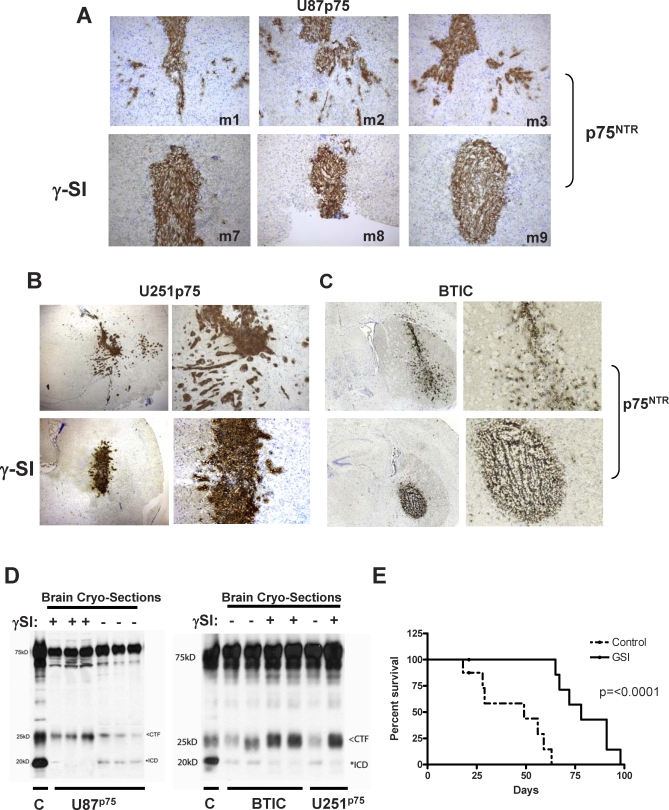
γ-Secretase Inhibitor Significantly Blocked p75^NTR^ Induced Glioma Migration and Invasion In Vivo (A–C) u87p75^NTR^ (A), U251p75^NTR^ (B), or p75^NTR^-positive BTICs established from a patient GBM specimen (C) were implanted intracerebrally into SCID mice (m1–m3 and m7–m9 represent individual mice). Three days (U87p75^NTR^ and U251p75^NTR^) or 5 d (BTIC) later, mice were administered s.c. 10 mg/kg γ-secretase inhibitor DAPT (γ-SI) or vehicle (corn oil) alone, once/day for 2–3 wk (three to five mice/group). The mice were sacrificed, and frozen brain sections were stained with an antibody against human p75^NTR^ (brown). Sections were counterstained with toluidine blue to visualize the cell nucleus (blue). Control animals given vehicle alone (corn oil; middle row) developed tumors with highly infiltrative edges (top panels). In marked contrast, animals that received daily injections of the γ-secretase inhibitor DAPT (bottom panels) developed localized tumors with demarcated edges. Similar results were seen in two independent experiments with three to five animals in each group. (D) γ-Secretase inhibitor blocks p75^NTR^ processing in vivo. Brain cryosections from three individual SCID mice implanted with u87p75^NTR^-, U251p75^NTR^-, or p75^NTR^-positive BTICs and administered s.c. 10 mg/kg γ-secretase inhibitor DAPT or vehicle (corn oil) alone were lysed in 2× loading buffer. Proteins were resolved on 10% SDS-PAGE gels and probed with a p75 cytoplasmic-specific antibody. In animals given vehicle alone, western blot analysis detected p75^NTR^-positive fragments migrating at 75, 24, and 19 kDa, whereas in cryosections from animals given the γ-secretase inhibitor DAPT, only the full-length p75^NTR^ and 25-kDa CTF were detected. U87 cells expressing p75^NTR^ and grown in the presence of epoxomicin (control; C) were used as a positive control. (The less than symbol [<] indicates CTF; the asterisk [*] indicates ICD.) (E) γ-Secretase inhibitor increases survival of animals bearing U87p75^NTR^ xenografts. Kaplan-Meier survival curves of SCID mice harboring U87p75^NTR^ intracranial tumors given s.c. injections of 10 mg/kg γ-secretase inhibitor DAPT or vehicle (corn oil) alone, once/day beginning on day 3. Animals given the γ-secretase inhibitor (GSI) survived significantly longer than control animals (*p* < 0.0001).

p75^NTR^ as a substrate for γ-secretase [26,33,39] adds to a growing list of proteins shown to be substrates for γ-secretase cleavage, including APP [52–54], Notch [55,56], and Notch ligands Delta1 and Jagged2 [57], ErbB4 [58], CD44 [59,60], and E-cadherin [61,62]. Our in vitro data and the recent application of γ-secretase inhibitors (egs.LY-450139 and LY-411575) in advanced clinical trials for Alzheimer disease [63–65] prompted us to investigate the use of γ-secretase inhibitors to treat highly invasive gliomas. Using an intracranial glioma model, we assessed the therapeutic potential of γ-secretase inhibitors. Parallel experiments were performed using the genetically distinct U87p75^NTR^ and U251p75^NTR^ glioma cell lines and the p75^NTR^-positive BTICs established from a patient GBM. Cells were implanted intracerebrally into SCID mice; and 3 d after implantation for U87p75^NTR^ and U251p75^NTR^ or 5 d after for BTICs, mice were administered subcutaneously (s.c.) either 10 mg/kg γ-secretase inhibitor or vehicle control (corn oil) once/day for 2–3 wk (three to five mice/group). Tumors were allowed to grow for a total of 4–6 wk, at which time, all animals were sacrificed, their brains removed, frozen in O.C.T. compound, and sectioned. Immunohistochemical staining of the frozen brain sections for anti-human nuclei (unpublished data) or p75^NTR^ (Figure 6A–6C) showed that animals implanted with U87p75^NTR^-, U251p75^NTR^-, or p75^NTR^-positive BTICs formed tumors with highly infiltrative edges (Figure 6A–6C; upper panels). In sharp contrast, animals implanted with U87p75^NTR^-, U251p75^NTR^-, or p75^NTR^-positive BTICs and given the γ-secretase inhibitor DAPT developed localized tumors with highly demarcated edges (Figure 6A–6C; lower panels). These results strongly suggested γ-secretase inhibition that results in blocking the generation of the p75^NTR^ ICD substantially inhibited the invasive ability of glioma cells in vivo. To establish whether the effects of the γ-secretase inhibition were confined to glioma invasion or had consequences on other biological functions, we assessed the effect of γ-secretase inhibition on glioma cell proliferation in vivo and found no significant change (Figure S5). The γ-secretase inhibitor had negliable effects on the self-renewal capability of the BTIC line used in Figure 5 (unpublished data).

To confirm that administration of DAPT inhibited p75^NTR^ ICD generation in vivo, 7–9 μm cryosections were lysed as described previously, and western blots for p75^NTR^ were performed (Figure 6D). These experiments show the presence of full-length p75^NTR^, CTF, and ICD in control animals that received s.c. administration of vehicle (corn oil) alone. In contrast, western blots of cryosections from animals that received daily injections of the γ-secretase inhibitor DAPT detected only the full-length and CTF fragments of p75^NTR^. Subcutaneous administration of γ-secretase inhibitor DAPT inhibited the generation of the p75ICD and resulted in a visible accumulation of the CTF. In addition and most importantly, animals bearing U87p75^NTR^ orthotopic xenographs and given daily s.c. injections of the γ-secretase inhibitor DAPT survived significantly longer (*p* < 0.0001) than control animals (Figure 6E), further highlighting the potential use of γ-secretase inhibitors in the clinical treatment of malignant glioma.

## Discussion

The p75^NTR^ signaling cascade is a complex signaling axis that depends on numerous factors, including cellular context and specific protein interactions that influence biological outcomes to regulated intramembranous proteolysis of p75^NTR^. For example, a recent report showed that in primary cultures of cerebellar neurons, p75^NTR^ ectodomain shedding and subsequent γ-cleavage is necessary for the growth inhibitory signal of myelin associated glycoprotein (MAG) [33]. Conversely, in retinal ganglion cells, neurotrophin-induced activation of p75^NTR^ was shown to promote neurite growth in a RIP-dependent manner [66]. Ligand-dependent induction of p75^NTR^ cleavage has also been reported in other cell systems, including sympathetic neurons and glial cells [28,30,34]. Similarly, we have shown that glioma migration is neurotrophin dependent [15] and that this neurotrophin-induced invasion is dependent on RIP of p75^NTR^.

To assess the role of RIP in p75^NTR^-mediated glioma invasion, we used a pharmacological and molecular approach both in vitro and in vivo to demonstrate that (1) p75^NTR^ proteolytic processing occurs in glioma cell lines, surgically resected tumor specimens, and BTICs isolated from patient specimens; (2) cleavage-resistant alleles of p75^NTR^ are insufficient to mediate glioma invasion; and (3) pharmacological inhibition with a clinically applicable γ-secretase inhibitor results in a dramatic decrease of glioma invasion both in vitro and in vivo and significantly prolonged survival of animals bearing p75^NTR^-positive intracranial tumors. Together, these data highlight the potential of using pharmacological inhibition to interfere with RIP as a therapeutic intervention for highly infiltrative p75^NTR^-positive gliomas.

One of the initial steps in regulating RIP is the shedding of the ECD by an α-secretase. This shedding event is required for subsequent cleavage of the CTF to generate an ICD. In order to show that both of these proteolytic events were important in the processing of p75^NTR^, we made a series of chimeric molecules with the Fas receptor, a related family member that does not generate CTF and ICD fragments [39,67]. The means by which the ECD is shed from the full-length p75^NTR^ protein and what its biological role is are not fully understood [68–70]. Glioma cells are known to express many proteases, including serine, cysteine, and metalloproteinases that are involved in invasion and tumor progression. The ADAM metalloproteinase disintegrins, including ADAM17 and ADAM10, have been described as prominent sheddases for p75^NTR^ as well as other transmembrane type-1 receptors such as APP [71–73], with recent in vivo evidence establishing a correlation with glioma invasion and an increase in ADAM17 under hypoxic stress [74,75]. The use of inhibitors targeting these proteinases may thus result in preventing RIP of p75^NTR^. Here, we have shown that the broad-range metalloproteinase inhibitor TAPI-2 was able to prevent both proteolytic processing of p75^NTR^ in glioma and p75^NTR^-mediated invasion. Although a possible therapeutic strategy for highly invasive p75^NTR^-positive tumors, previous clinical attempts to inhibit the protease-rich environment of tumors using broad-spectrum MMP inhibitors have so far proven to be ineffective as anti-cancer agents, with phase II and III trials failing to show efficacy or survival benefit [76,77]. The reason for this lack of efficacy may result in part from the fact that glioblastomas produce high levels of proteases, many of which have been suggested to help facilitate tumor cell survival and invasiveness [74,78–83]. Attempts, therefore, to inhibit the ectodomain shedding of p75^NTR^ in a clinical setting may prove difficult.

The second proteolytic event with possible direct therapeutic importance is mediated by the γ-secretase complex, which is composed of several proteins including presenilin, nicastrin, APH-1, and presenilin enhancer 2 (PEN-2) [84]. This protein complex is known to be essential in the normal processing of amyloid β-peptides from β-APP. Abnormal accumulation of amyloid β-peptides with the formation of plaques is believed to be the pathogenesis of Alzheimer disease. Given the connection between Alzheimer disease and γ-secretase, there has been great interest in developing compounds that can inhibit this protein complex with some of these compounds already in phase II/III clinical trials [85]. The exact molecular mechanism(s) by which the ICD fragment of p75^NTR^ exerts the invasive behavior of glioma cells is unknown. As is the case with the Notch signaling pathway [41,86,87], there have been recent studies to suggest that the ICD fragment can translocate to the nucleus, but whether it acts as part of a transcriptional complex is unclear [28,30]. In addition, myelin-associated glycoprotein binding to cerebellar neurons induces α- and γ-secretase proteolytic cleavage of p75^NTR^, and the resulting ICD fragment is necessary for both the activation of the small molecular weight GTPase, RhoA, and inhibition of neurite outgrowth [33]. Whether these processes or others are regulated by the p75^NTR^ ICD fragment in glioma cells remains to be determined.

In our present study, we show that neurotrophin-induced p75^NTR^ proteolytic processing is required for p75^NTR^-mediated glioma invasion in vitro and in vivo. Furthermore, daily administration of the γ-secretase inhibitor DAPT to animals bearing p75^NTR^-postive intracranial tumors significantly prolonged survival. These results are intriguing and support the possible clinical application of γ-secretase inhibitors for the treatment of these deadly tumors. We cannot, however, exclude the possibility that we are inhibiting the processing of other proteins that may be involved in glioma invasion since γ-secretase is known to mediate the proteolytic processing of several transmembrane proteins [52,53,55,57–61]. The biochemical evidence presented here, however, supports the hypothesis that the anti-invasive effect of γ-secretase inhibition is at least in part the result of inhibition of p75^NTR^ RIP. Moreover, the fact that we did not observe any significant effects on proliferation or survival of the human glioma cells in vivo suggests that the dominant mechanism of activity is the inhibition of p75^NTR^-mediated glioma invasion.

Excitingly, we also found that a large percentage (four out of five) BTICs from primary glioma patient tissue express high levels of p75^NTR^. This rare population of cells with stem-like properties and the ability to repopulate the tumor [3–6] have been shown to be resistant to our current therapies (radiation and temozolomide) and thus may represent a “disease reservoir” for these devastating tumors [88,89]. Unlike the U87 parental cells, these cells are highly invasive in vivo, and treatment with a γ-secretase inhibitor dramatically blocked their invasive nature (Figure 6). Several recent studies have demonstrated strong similarities between BTICs and neural stem and progenitor cells [4,90,91]. However, whether human glioblastoma stem cells arise from mutated neural stem cells or a more mature cell type that acquires self-renewal capacity remains to be determined. Interestingly, a small population of cells (0.3%) within the stem cell niche of the adult rat subventricular zone has neurosphere-forming capacity, express p75^NTR^ [92], and appear to be maintained from birth through adulthood [93–95]. In addition, the more migratory p75^NTR^ glioma cell population in clinical glioblastoma patient specimens also represents a small percentage of the main tumor mass [15]. It is intriguing to note that glioma cells that express high levels of p75^NTR^ seem to possess many characteristics of BTICs, including self-renewal, extensive brain parenchymal migration, and potential for differentiation (J. J. P. Kelly and S. Weiss, unpublished data). Whether p75^NTR^ is an early brain tumor stem cell marker, at least for some GBMs, remains to be determined.

In a previous study, we postulated that p75^NTR^ itself may be a valid target for the treatment of glioma, and now we propose that abrogation of the cellular processing of p75^NTR^ represents an additional therapeutic target. Although these inhibitors may have application in malignant glioma, they may have an even broader application for cancer, as p75^NTR^ has also been implicated in other cancers, including melanoma, specifically the more aggressive melanomas that metastasize to the brain [96–98]. Thereby, therapies that target the processing of p75^NTR^ may also be beneficial for other metastatic cancers.

## Materials and Methods

### Cell culture.

The human glioma cell lines U87, U118, and U343 were obtained from the American Type Culture Collection. The human glioma cell line U251N was a kind gift from V. W. Yong (University of Calgary, Calgary, Alberta, Canada). All cells were maintained in complete media (Dulbecco's modified eagle's medium [DMEM] F12 supplemented with 10% heat-inactivated fetal bovine serum [FBS], 0.1 mM nonessential amino acids, 2 mM l-glutamine, and 1 mM sodium pyruvate [Gibco BRL, http://www.invitrogen.com]) at 37 °C in a humidified 5% CO_2_ incubator. Cells were passaged by harvesting with trypsin (Gibco BRL) at 80%–90% confluence. Stable transfectants of U87, U251, U118, and U343 cells were maintained in identical media with the exception of the addition of 400 μg/ml of G418 (Invitrogen, http://www.invitrogen.com).

### Construction of p75^NTR^ cleavage-resistant plasmids.

The human p75^NTR^ expression vector was constructed as described previously [98]. The expression plasmids containing the p75^NTR^ mutants were constructed by subcloning of PCR fragments containing the desired p75^NTR^ sequences. Chimeric proteins were created by replacing either the transmembrane (*p75FasTM*) or the extracellular stalk domain of p75^NTR^ (*p75FasS*) with equivalent domains from the Fas receptor [Figure 3C] as described by Domeniconi et al. 2005 [33]. The neurotrophin-binding mutant that was mutated by a four-amino acid (ARRA) insertion after amino acid residue130 was termed *p75CRD130* [15,42,43,47]. The p75^NTR^ intracellular domain construct was created using amino acids 236–399 of the wild-type receptor plus a methionine at the amino terminus (p75-ICD). The original p75^NTR^ templates were from B. Hempstead (p75WT; Weill Medical College of Cornell University, New York, New York) and M. Chao (pT3/T7-p75; New York University School of Medicine, New York, New York). All constructs were inserted into pcDNA 3.1 expression vectors (Invitrogen). The sequences of all the mutant expression plasmids were confirmed prior to stable transfection.

### Transfection of glioma cell lines.

Transfection of glioma cell lines was performed as described previously [15]. Briefly, cells to be transfected were seeded at 2 × 10^5^ cells/well in six-well plates, and incubated at 37 °C overnight. Vector DNA was introduced to the cells using FuGENE 6 transfection reagent (Roche Diagnostic, http://www.roche.com) according to the manufacturer's instructions. The following day, the medium was changed to fresh complete medium containing the antibiotic G418 (concentration determined by toxicity curve for each cell line) to select for those cells that had taken up the vector. The cells were then grown under antibiotic selection until the cells were at confluence. For U87p75^NTR^, U251p75^NTR^, U118p75^NTR^, U343p75^NTR^, U87p75FasTM, U87p75FasS, U87p75CRD130, U251p75FasTM, and U251p75FasS transfection, transfected cells were identified by flow cytometry and western blot.

### Western blotting.

The desired cells were washed in ice-cold PBS and lysed by gentle rocking in lysis buffer (50 mM Tris-HCl [pH 7.4], 150 mM NaCl, 10 mM NaF, 0.02% NaN_3_, 0.5% sodium deoxycholate, 0.1% SDS, 1% Nonidet P-40, 1% Triton X-100, 1 mM EDTA, 60 mM β-octyl glucoside, 25 μg/ml aprotinin, 10 μg/ml leupeptin, 3 mM sodium orthovanadate, 1 mM PMSF) at 4 °C. Cellular debris was removed by centrifugation, and protein quantification was performed using the bicinchoninic acid (BCA) assay (Pierce Biotechnology, http://www.piercenet.com). Proteins were resolved on 12% SDS-PAGE gels, and western blots were performed using the following primary antibodies: rabbit polyclonal anti-human p75^NTR^ intracellular domain (Promega, http://www.promega.com), mouse monoclonal anti-human p75^NTR^ ECD (Upstate Biotechnology), mouse monoclonal anti-β-tubulin (Sigma-Aldrich, http://www.sigmaaldrich.com), or mouse monoclonal anti-β-actin (Cell Signaling Technology, http://www.cellsignal.com). The appropriate HRP-conjugated secondary antibody (Pierce Biotechnology) was used, and blots were visualized using enhanced chemiluminescence (Amersham Biosciences).

### Flow cytometric analysis of p75^NTR^.

A total of 1 × 10^6^ cells stably transfected with p75^NTR^ wild type or p75^NTR^ cleavage-resistant constructs *p75FasTM* and *p75FasS* were collected using Puck's EDTA at 37 °C and then washed in PBS containing 1 mM EDTA (PBS/EDTA). Cells were exposed to the monoclonal anti-p75^NTR^, clone ME20.4 (which recognizes the extracellular domain; Upstate Biotechnology, http://www.upstate.com), diluted 1:250 in PBS/EDTA for 30 min on ice. Cells transfected with pcDNA vector alone were used as negative controls. After washing with PBS/EDTA, cells were incubated with Alexa-488 conjugated goat anti-mouse IgG (Invitrogen/Molecular Probes, http://www.probes.invitrogen.com) diluted 1:500 in PBS/EDTA for 30 min on ice. Cells were then washed with PBS/EDTA, resuspended in PBS/EDTA, and analyzed using a FACScan flow cytometer (Becton, Dickinson and Company, http://www.bdbiosciences.com).

### ELISA.

U87 and U251 glioma cells stably transfected with p75^NTR^ wild type or the cleavage-resistant constructs were allowed to condition culture medium for 5 d. The conditioned medium was then collected, centrifuged, and filtered through a 0.2-μm syringe filter (VWR International, http://www.vwr.com). The remaining cells were washed with ice-cold PBS, and total cellular lysates were extracted as described for western blot. Protein quantification was performed using the BCA assay (Pierce Biotechnology), and BDNF, nerve growth factor (NGF), or neurotrophic factor 3 (NT-3) ELISA (R&D Systems, http://www.rndsystems.com) was performed as per the company protocol. Briefly, MaxiSorp ELISA plates (Nalge Nunc International, http://www.nalgenunc.com.com) were coated with monoclonal anti-human BDNF, NGF, or NT-3 (R&D Systems), nonspecific binding was blocked, and then the standards of serial dilutions of recombinant human BDNF, NGF, or NT-3 (Sigma-Aldrich) and equal volumes of conditioned medium or equal quantities of lysate were added. Bound antigen was detected using the corresponding biotinylated antibody, streptavidin HRP, and a tetramethylbenzidine substrate (R&D Systems). Absorbance was measured at 450 nm.

### In vitro p75^NTR^ proteolysis.

For in vitro p75^NTR^ cleavage assessment, the desired cells were treated for 4 h at 37 °C and 5% CO_2_ with the proteasome inhibitor epoxomicin (1 μM) (Calbiochem, http://www.emdbiosciences.com) and/or γ-secretase inhibitor Compound X (2 μM) (Calbiochem). DMSO was used as the vehicle control. Cells were then washed one time with cold PBS on ice, lysed in lysis buffer (50 mM Tris-HCl [pH 7.4], 150 mM NaCl, 10 mM NaF, 0.02% NaN_3_, 0.5% sodium deoxycholate, 0.1% SDS, 1% Nonidet P-40, 1% Triton X-100, 1 mM EDTA, 60 mM β-octyl glucoside, 25 μg/ml aprotinin, 10 μg/ml leupeptin, 3 mM sodium orthovanadate, 1 mM PMSF) at 4 °C with protease inhibitors, and centrifuged for 5 min at 14,000×*g*; supernatants were quantified by BCA assay (Pierce Biotechnology) for use in SDS-PAGE. The CTF (24 kDa) and ICD (19 kDa) fragments were detected using an antibody specific to the p75^NTR^ ICD (Promega).

### Circular Monolayer Migration Assay.

Migration assays were performed using a microliter-scale radial monolayer migration assay as described previously [15]. Briefly, ten-well Teflon-masked microscope slides were coated with 20 μg/ml laminin, followed by the addition of 50 μl of medium to each well. Sedimentation manifold (Creative Scientific Methods, http://www.creative-sci.com) was placed over the laminin-coated slide. Cells were seeded through the central lumen of the cell sedimentation cylinder at 2,000 cells/well (five wells per cell type/condition) to establish a circular 1-mm diameter confluent monolayer. Slides were placed on ice for 60 min and then incubate at 37 °C for approximately 6 h. Attachment of the cells was confirmed prior to removing the sedimentation manifold. Once the sedimentation manifolds were removed, cells were given complete medium containing the γ-secretase inhibitor (Compound X, 2 μM). A digital image of the cells was taken (before migration = 0 h) using a Zeiss Axiovert 200M inverted fluorescent microscope (Carl Zeiss, http://www.zeiss.com). The cells were then incubated in a humidified chamber at 37 °C and 5% CO_2_, and a second digital image was taken 48 h later. Best-fit circles were drawn around the area covered by the cells at the 0 h and 48 h time points, and the actual cell area was determined using Axiovision 4.2 imaging software (Carl Zeiss). Quantitative migration scores were calculated as the increase in the area covered by the cells beyond the initial area of the cells.

### 3D-invasion assay.

To test the invasive ability of the p75^NTR^ cleavage-resistant constructs, actively growing glioma cells U87 and U251 stably transfected with p75^NTR^ and p75^NTR^ cleavage-resistant constructs were suspended in a collagen gel solution and plated in transwell chambers with 8.0-μm pore size polycarbonate membrane (Costar, http://www.costar.com). The collagen gel was prepared by mixing collagen solution (Chemicon International 3D Collagen cell culture system, Cat# ECM675, http://www.chemicon.com) with 5× DMEM F12 medium on ice. Neutralization solution (40:1) and extracellular matrix (ECM) proteins were added at a concentration of 10 μg/ml (laminin, fibronectin, chondroitin sulfate proteoglycan, Chemicon). A total of 1 × 10^5^ cells were suspended in 350 μl of collagen gel solution, and 70 μl of the collagen/cell mixture was pipetted into the Transwell chambers (five chambers for each cell line). Chambers were immediately transferred to a 37 °C incubator for 60 min to allow the matrix to polymerize. Once polymerized, 100 μl of serum-free DMEM was added to the upper chamber and 1.0 ml of 10% FBS complete medium with the γ-secretase inhibitor Compound X (2 μM) was added to the lower chamber. Transwell chambers were kept at 37 °C for 6 h, at which time the chamber was washed with PBS, fixed with acid-alcohol for 15 min at room temperature, and then stained with hematoxylin. Any cells remaining in the top chamber were removed, and membranes were mounted on glass slides. Four different fields were counted for each membrane.

### Animals.

Six- to 8-wk-old female SCID mice were purchased from Charles River Laboratories (http://www.criver.com). The animals were housed in groups of three to five and maintained on a 12-h light/dark schedule with a temperature of 22 °C ± 1 °C and a relative humidity of 50% ± 5%. Food and water were available ad libitum. All procedures were reviewed and approved by the University of Calgary Animal Care Committee.

### In vivo studies using an intracranial glioma model.

Actively growing glioma cells stably transfected with p75^NTR^ and p75^NTR^ cleavage resistant constructs were harvested by trypsinization, washed, and resuspended in sterile PBS(137 mM NaCl, 8.1 mM Na_2_HPO_4_, 2.68 mM KCl, and 1.47 mM KH_2_PO_4_ [pH 7.5]). These cells were implanted intracerebrally into the right putamen of SCID mice (1 × 10^5^ cells/mouse) at a depth of 3 mm through a scalp incision and a 0.5-mm burr hole made 1.5–2 mm right of the midline and 0.5–1 mm posterior to the coronal suture. All mice were anaesthetized by intraperitoneal administration of ketamine (85 mg/kg) plus xylazine (15 mg/kg) (MTC Pharmaceuticals). The stereotactic injection used a 5-μl syringe (Hamilton Co., www.hamiltoncompany.com) with a 30-g needle mounted on a Kopf stereotactic apparatus (Kopf Instruments). After withdrawal of the needle, the incision was sutured. Animals were sacrificed at specific time points (generally weekly, from 2–6 wk postinjection) or when they lost 20% of their body weight or had difficulty ambulating, feeding, or grooming. For some experiments, BrdU was given by intraperitoneal injection 24 h prior to sacrifice. Following sacrifice, the brains were removed, frozen in Tissue-Tek O.C.T. compound (Electron Microscopy Sciences, http://www.emsdiasum.com), and cryo-sectioned into 7–9-μm sections for examination by immunohistochemistry and in vivo p75^NTR^ proteolysis assessment.

### Immunohistochemistry.

Frozen sections were air-dried at room temperature, fixed with cold acetone, and then rinsed with PBS. Endogenous peroxidases in the sections were inactivated with 0.075% H_2_O_2_/methanol, and nonspecific binding was blocked with 10% normal goat serum in PBS. The sections were incubated with rabbit polyclonal anti-human p75^NTR^ ICD antibody (Promega) or mouse monoclonal anti-human nuclei (Chemicon) in blocking buffer overnight at 4 °C. Following washing with PBS, the appropriate biotinylated secondary antibody (Vector Laboratories, http://www.vectorlabs.com) was applied. Avidin-biotin peroxidase complexes were then formed using the VECTASTAIN Elite ABC kit (Vector Laboratories) and detected by addition of SIGMAFAST DAB (3,3′-diaminobenzidine tetrahydrochloride) (Sigma-Aldrich), which was converted to a brown reaction product by the peroxidase. Toluidine blue (for frozen sections) was used as a nuclear counterstain. Sections were then dehydrated in an ethanol/xylene series and mounted with Entellan (Electron Microscopy Sciences).

### In vivo p75^NTR^ proteolysis.

For detection of p75^NTR^ proteolytic processing in vivo, the desired cells were implanted intracerebrally into SCID mice as described previously. Mice were sacrificed 3–4 wk later. Following sacrifice, the brains were removed, frozen in Tissue-Tek O.C.T. compound (Electron Microscopy Sciences), cryosectioned into 7–9-μm sections, and alternating sections were stained with toluidine blue. Based on the size of tumor, cryosections were lysed in 2× loading buffer (0.1 M Tris-HCl [pH 6.8], 4% SDS, 20% glycerol, 10% β-mercaptoethanol, 0.02% bromphenol blue). Proteins were resolved on 12% SDS-PAGE gels, and western blots were performed using an anti-p75^NTR^ cytoplasmic specific antibody (Promega).

### Detection of p75^NTR^ proteolysis in patient specimens.

Tumor and normal tissues were obtained from the Canadian Brain Tumor Tissue Bank in London, Ontario, and the Brain Tumor Tissue Bank at the Clark Smith Brain Tumor Center within the Southern Alberta Cancer Research Institute. Briefly, tissue was taken during surgery while patients were under a general anesthetic, and was placed immediately in liquid nitrogen and stored at −80 °C. An institutional ethics board approved the collection and use of all of the surgical tissue used, and all of the patients gave signed informed consent. Frozen sections of patient tumor tissue were lysed in lysis buffer (50 mM Tris-HCl [pH 7.4], 150 mM NaCl, 10 mM NaF, 0.02% NaN_3_, 0.5% sodium deoxycholate, 0.1% SDS, 1% Nonidet P-40, 1% Triton X-100, 1 mM EDTA, 60 mM β-octyl glucoside, 25 μg/ml aprotinin, 10 μg/ml leupeptin, 3 mM sodium orthovanadate, 1 mM PMSF) with protease inhibitors on ice using a homogenizer (Life Technologies). Lysates were centrifuged for 5 min at 14,000×*g* to remove debris, and supernatants were quantified by BCA assay (Pierce Biotechnology). In this study, based on our previous immunohistochemistry results of p75^NTR^ expression in GBM patient specimens, western blot analysis was performed on nine GBMs and five mid-grade glioma samples using an antibody specific for the intracellular domain of p75^NTR^.

### Primary culture of brain tumor-initiating cells (BTICs).

Tumor and normal tissues were obtained from the Tumor Tissue Bank in Foothills Hospital, Calgary, Alberta. Operative samples of human gliomas were obtained during brain tumor surgery and transported to the laboratory in serum-free DMEM-F12. Primary cultures of brain tumor-initiating cells (BTICs) were established. Briefly, necrotic and connective tissue and any blood clots were removed using forceps, and the remaining tissue was washed in PBS and cut into pieces of approximately 1 mm^3^. The tissue was then incubated for 5–10 min at 37 °C in an enzyme cocktail of trypsin (0.25%) and DNase I (10 μg/ml) in PBS. The digested tissue was strained through a 100-μm mesh and washed with PBS. Following lysis of the red blood cells, the remaining cells were washed with PBS and strained through a 40-μm mesh. After spinning at 1,000 rpm for 5 min, cells were resuspended in stem cell medium (M medium) or stem cell medium plus EGF and bFGF (EF medium). M medium is NeuroCult NS-A basal medium (human) 450 ml plus NeuroCult NS-A Proliferation Supplements (human) 50 ml (StemCell Technologies). EF media is M media plus human recombinant EGF (20 ng/ml; Sigma) and bFGF (20 ng/ml; Chemicon).

### Immunocytochemistry of BTICs.

Eight-well LAB-TEK chamber slides (Nalgel Nunc, http://www.nuncbrand.com) were coated with poly-l-ornithine (Sigma) and incubated at 37 °C for 1 h. The desired BTICs were plated into chambers with stem cell culture medium to equilibrate overnight at 37 °C, 5% CO_2_. Chambers were then rinsed with PBS, fixed in 3.7% paraformaldehyde diluted in PBS for 20 min, and rinsed twice with PBS. Anti-p75^NTR^ cytoplasmic specific antibody (1:3,000, Promega) and antibodies to the progenitor markers: Nestin (1:1,000), hSOX2 (1:5,000), and mushashi (1:200) (R&D System) were diluted in 0.3% Triton X-100/PBS/10% goat serum, and 200 μl of these solutions were added to each chamber, incubated overnight at 4 °C. Following washing with PBS, the appropriate Cy-3– or FITC-conjugated secondary antibodies (1:2,000) (Cedarlane, http://www.cedarlanelabs.com) were applied and incubated for 30 min in the dark at room temperature. The chambers were removed, and slides were mounted with DAPI counterstained mounting medium (Vector Laboratories) and imaged with an Olympus IX70 Delta Vision RT microscope and the SoftWoRx software package.

### Detection of p75^NTR^ proteolysis in BTICs.

Based on the immunocytochemistry staining results, BTICs were collected, dissociated by polished glass pipette, aliquoted into six-well plates, and treated with the proteasome inhibitor epoxomicin (1 μM) (Calbiochem) and/or the γ-secretase inhibitor Compound X (2 μM) (Calbiochem), or DMSO vehicle alone, for 4 h at 37 °C and 5% CO_2_. Cells were then washed twice with cold PBS on ice, lysed in lysis buffer with protease inhibitors, and quantified by BCA assay. Western blots for p75^NTR^ using a p75^NTR^ cytoplasmic-specific antibody were performed.

### In vivo γ-secretase studies using intracranial glioma models.

Actively growing glioma cell line U87p75 and BTICs were implanted intracerebrally into SCID mice as described previously. Three days later for the U87p75 cell line, and 5 d later for BTICs, mice were administered s.c. vehicle (corn oil) or 10 mg/kg γ-secretase in corn oil once/day for 2 wk (U87p75^NTR^) or for 3 wk (BTIC). At 3 wk for animals bearing U87p75^NTR^ tumors or 4 wk for animals bearing BTIC tumors, animals were sacrificed, the brains were removed, frozen in Tissue-Tek O.C.T. compound, and cryosectioned into 7–9-μm sections. The cryosections were used for tumor immunohistochemistry staining and for assessment of in vivo p75^NTR^ cleavage. For survival studies (eight animals per group), U87p75^NTR^ glioma cells were implanted intracerebrally into SCID mice. Animals were given daily s.c. injections of 10 mg/kg γ-secretase inhibitor DAPT or vehicle (corn oil) alone, once/day beginning on day 3. Animals were followed until sacrifice was required.

### Statistical analysis.

Statistical analysis of data was performed using GraphPad Prism software (GraphPad Software). Survival curves were generated using the Kaplan-Meier method. The log-rank test was used to compare the distributions of survival times. A *p-*value of less than 0.05 was considered statistically significant.

## Supporting Information

Figure S1 TNF-α Protease Inhibitor (TAPI)-2 Prevents the Proteolytic Processing of p75^NTR^ and Also Decreases the Migration of U87-p75^NTR^ Cells (A) A total of 7.5 × 10^5^ U87p75 or U87pcDNA cells were plated in collagen III-coated six-well plates and treated with either normal growth medium or medium supplemented with TAPI-2 at 20 μM overnight. Western blots for p75^NTR^ were probed with an antibody specific to the cytoplasmic domain of p75^NTR^, which detects full-length (75 kDa), CTF (indicated by the less than symbol [<]; 25 kDa) and ICD (indicated by an asterisk [*]; 19 kDa) peptides. In cell lysates from U87p75 glioma cells, peptides corresponding to the full-length, CTF, and ICD fragments were detected, whereas only the full-length p75^NTR^ receptor was detected in cells treated with TAPI-2.
(B) A total of 5 × 10^4^ U87p75 or U87pcDNA cells were plated into the upper chambers of Transwell plates coated with brain-like matrix (collagen III with plasma fibronectin, chondroitin sulfate proteoglycans, and laminins added as minor components). The cells were treated with either normal growth medium, or medium supplemented with 20 μM TAPI-2 for 4 h. Cells were fix/stained with 1% crystal violet in ethanol, and the cells on the under side of the membrane (migrated cells) were counted by light microscopy (*n* = 7; double asterisks [**] indicate *p* = 0.019).
(258 KB PPT)

Figure S2 γ-Secretase Inhibition Does Not Effect Proliferation or Survival of p75^NTR^-Expressing U87 Glioma Cells. Inhibition of γ-secretase does not effect proliferation or survival of the highly invasive human glioma cell lines U87R isolated by serial in vivo selection or the U87p75 cells, which ectopically express p75^NTR^. U87R and U87p75 were treated with and without 2 μM γ-secretase inhibitor Compound X (CompX), and survival and proliferation were assessed at 72 h by MTT assay (A) and crystal violet proliferation assay (B and C). The results show that 2 μM γ-secretase inhibitor CompX does not effect survival (A) or proliferation (B and C) of U87R and U87p75 (*p* > 0.05).
(365 KB PPT)

Figure S3 Biochemical Characterization of U87 and U251 Glioma Cells Expressing p75^NTR^ Wild Type or Cleavage-Resistant Chimeric Alleles (A) Expression and topography of the full-length, chimeric constructs (*p75FasTM* and *p75FasS*) and the neurotrophin-binding mutant *p75CRD130* at the plasma membrane were confirmed by flow cytometric analysis using a p75^NTR^ extracellular domain-specific antibody. U87pcDNA and U251pcDNA glioma cells were used as controls.
(B–D) The p75^NTR^ cleavage-resistant constructs retain their ability to bind ligand (C and D). Conditioned medium (B) and total cell lysates (C) from U87 and U251 cells expressing full-length p75, *p75FasTM*, *p75FasS*, and *p75CRD130* were analyzed by ELISA for their ability to bind BDNF. The p75^NTR^-negative U87pcDNA and U251pcDNA were used for comparison. Expression of p75^NTR^ full-length or the cleavage-resistant chimeric proteins (*p75FasTM* and *p75FasS*) produced a shift in BDNF localization from the conditioned medium to the cell lysate, consistent with the binding of BDNF to p75^NTR^. In contrast, cells expressing the ligand-binding mutant *p75CRD130* did not produce a shift in BDNF localization in accordance with the in ability to bind ligand. Values shown are the mean ± standard error of the mean (s.e.m.) for a single experiment. Similar results were seen in three independent experiments; triple asterisks (∗∗∗) indicate *p* < 0.001 as compared to pcDNA control for each cell line (one-way ANOVA with the Neuman-Keuls post-test).
(442 KB PPT)

Figure S4 Proteolytic Processing of p75^NTR^ Mutant Chimeras U87 (left panel) and U251 (right panel) stably transfected with p75^NTR^ wild type, the cleavage-resistant chimeras (*FasS* and *FasTM*), or the neurotrophin-binding mutant *CRD130* were treated with the proteasome inhibitor epoxomicin (Epo, 2 μM) and/or the specific inhibitor of γ-secretase, Compound X (CompX, 2 μM) for 4 h. Western blots for p75^NTR^ were probed with an antibody specific to the cytoplasmic domain of p75^NTR^ which detects full-length (75 kDa), CTF (indicated by the less than symbol [<]; 25 kDa), and ICD (indicated by the asterisk [*]; 19 kDa) peptides. In cell lysates from glioma cells expressing *p75FasTM* chimera, only the 24-kDa fragment was detected, whereas the *p75FasS* chimera and the *p75CRD130* ligand binding site mutant did not display any cleaved products.
(1.30 MB PPT)

Figure S5 γ-Secretase Inhibition Does Not Effect Glioma Cell Proliferation In Vivo u87p75^NTR^- (A), U251p75^NTR^-, (B) or p75^NTR^-positive BTICs established from a patient GBM specimen (C) were implanted intracerebrally into SCID mice. Three days (U87p75^NTR^ and U251p75^NTR^) or 5 d (BTIC) later, mice were administered s.c. 10 mg/kg γ-secretase inhibitor DAPT or vehicle (corn oil) alone, once/day for 2–3 wk (three to five mice/group). Bromodeoxyuridine (BrdU) was injected into the tumor-bearing mice 24 h prior to their sacrifice. Frozen brain sections were stained with an antibody against BrdU and counterstained with toluidine blue to visualize the cell nucleus. Cells that had divided during the 24 h prior to sacrifice stained positively for BrdU, and the percentage of BrdU-positive cells were counted. Bar graph represents the percentage of BrdU-positive cells in five consecutive fields. The γ-secretase inhibitor DAPT had no significant effect on proliferation in vivo of the p75^NTR^-glioma cells as compared to their untreated controls.
(60 KB PPT)

## Abbreviations

ADAM: a disintegrin and metalloprotease
APP: amyloid precursor protein
BTIC: brain tumor-initiating cell
BDNF: brain-derived neurotrophic factor
CTF: C-terminal fragment
ECD: extracellular domain
EGF: epidermal growth factor
GBM: glioblastoma multiforme
ICD: intracellular domain
p75NTR: p75 neurotrophin receptor
RIP: regulated intramembrane proteolysis
s.c.: subcutaneous
s.e.m.: standard error of the mean
TAPI: tumor necrosis factor-α protease inhibitor
TNF: tumor necrosis factor
