# Supplementary figures and images for: Gamma-Secretase Represents a Therapeutic Target for the Treatment of Invasive Glioma Mediated by the p75 Neurotrophin Receptor
Source: PLoS Biol. 2008 Nov 25;6(11):e289. doi: 10.1371/journal.pbio.0060289 (PMC2586378; doi:10.1371/journal.pbio.0060289)

## Slide 1
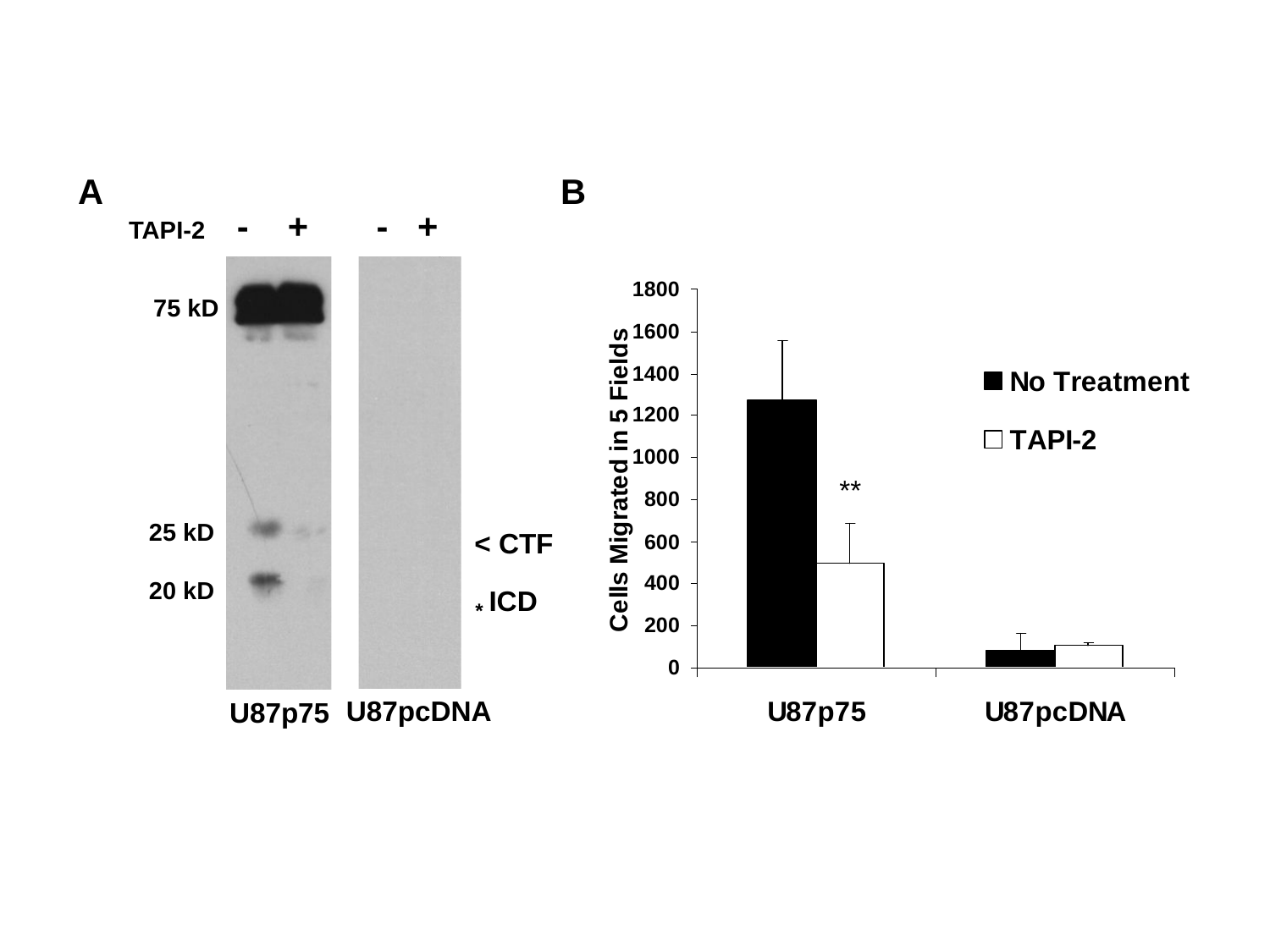

A
B
TAPI-2 - + - +
75 kD
**
25 kD
< CTF
20 kD
* ICD
U87pcDNA
U87p75

Supplement: Figure S1 — (A) A total of 7.5 × 10^5^ U87p75 or U87pcDNA cells were plated in collagen III-coated six-well plates and treated with either normal growth medium or medium supplemented with TAPI-2 at 20 μM overnight. Western blots for p75NTR were probed with an antibody specific to the cytoplasmic domain of p75NTR, which detects full-length (75 kDa), CTF (indicated by the less than symbol [<]; 25 kDa) and ICD (indicated by an asterisk [*]; 19 kDa) peptides. In cell lysates from U87p75 glioma cells, peptides corresponding to the full-length, CTF, and ICD fragments were detected, whereas only the full-length p75NTR receptor was detected in cells treated with TAPI-2. (B) A total of 5 × 10^4^ U87p75 or U87pcDNA cells were plated into the upper chambers of Transwell plates coated with brain-like matrix (collagen III with plasma fibronectin, chondroitin sulfate proteoglycans, and laminins added as minor components). The cells were treated with either normal growth medium, or medium supplemented with 20 μM TAPI-2 for 4 h. Cells were fix/stained with 1% crystal violet in ethanol, and the cells on the under side of the membrane (migrated cells) were counted by light microscopy (n = 7; double asterisks [**] indicate p = 0.019). (258 KB PPT) [file pbio.0060289.sg001.ppt]

## Slide 1
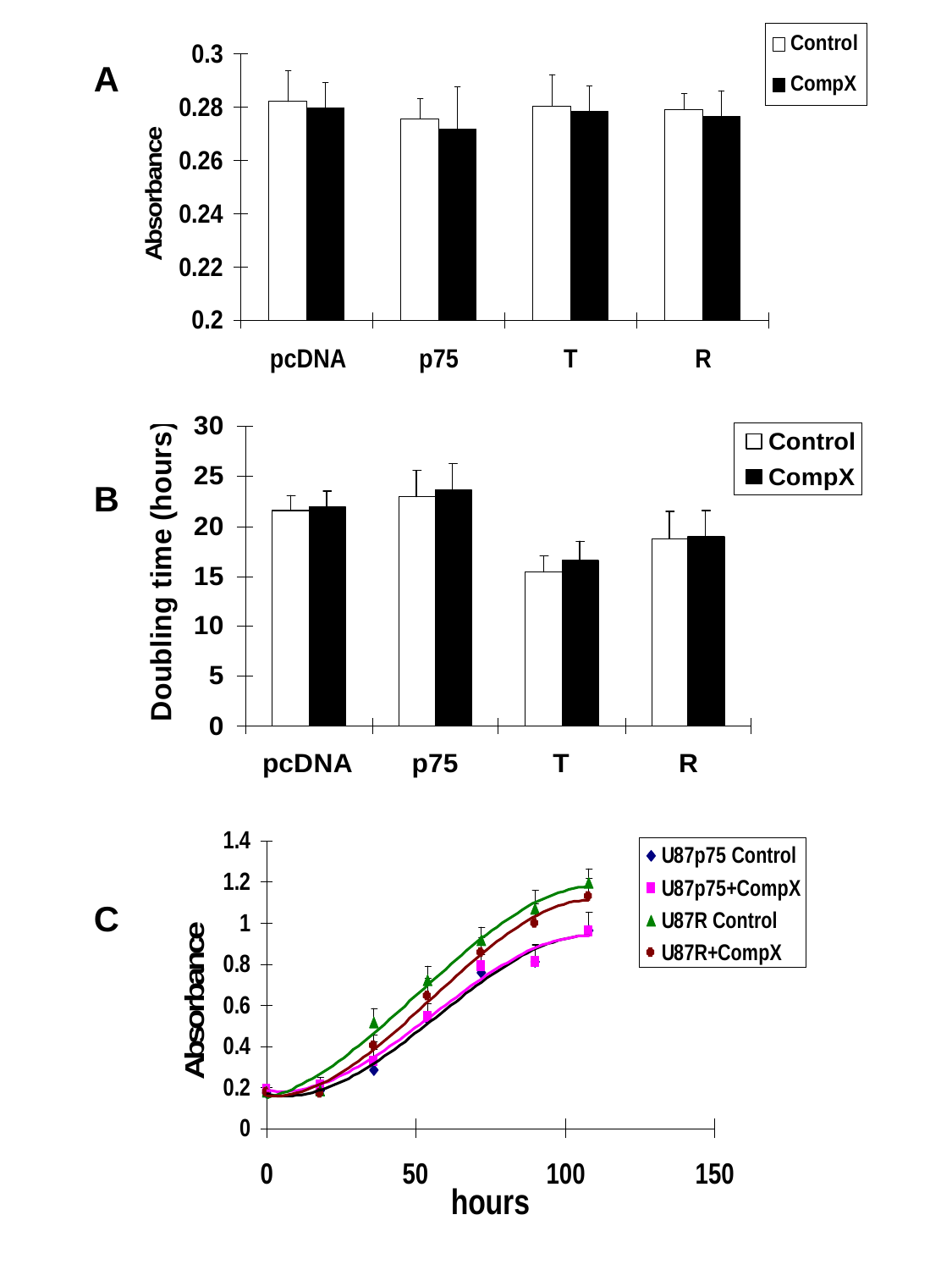

A
B
C

Supplement: Figure S2 — Inhibition of γ-secretase does not effect proliferation or survival of the highly invasive human glioma cell lines U87R isolated by serial in vivo selection or the U87p75 cells, which ectopically express p75NTR. U87R and U87p75 were treated with and without 2 μM γ-secretase inhibitor Compound X (CompX), and survival and proliferation were assessed at 72 h by MTT assay (A) and crystal violet proliferation assay (B and C). The results show that 2 μM γ-secretase inhibitor CompX does not effect survival (A) or proliferation (B and C) of U87R and U87p75 (p > 0.05). (365 KB PPT) [file pbio.0060289.sg002.ppt]

## Slide 1
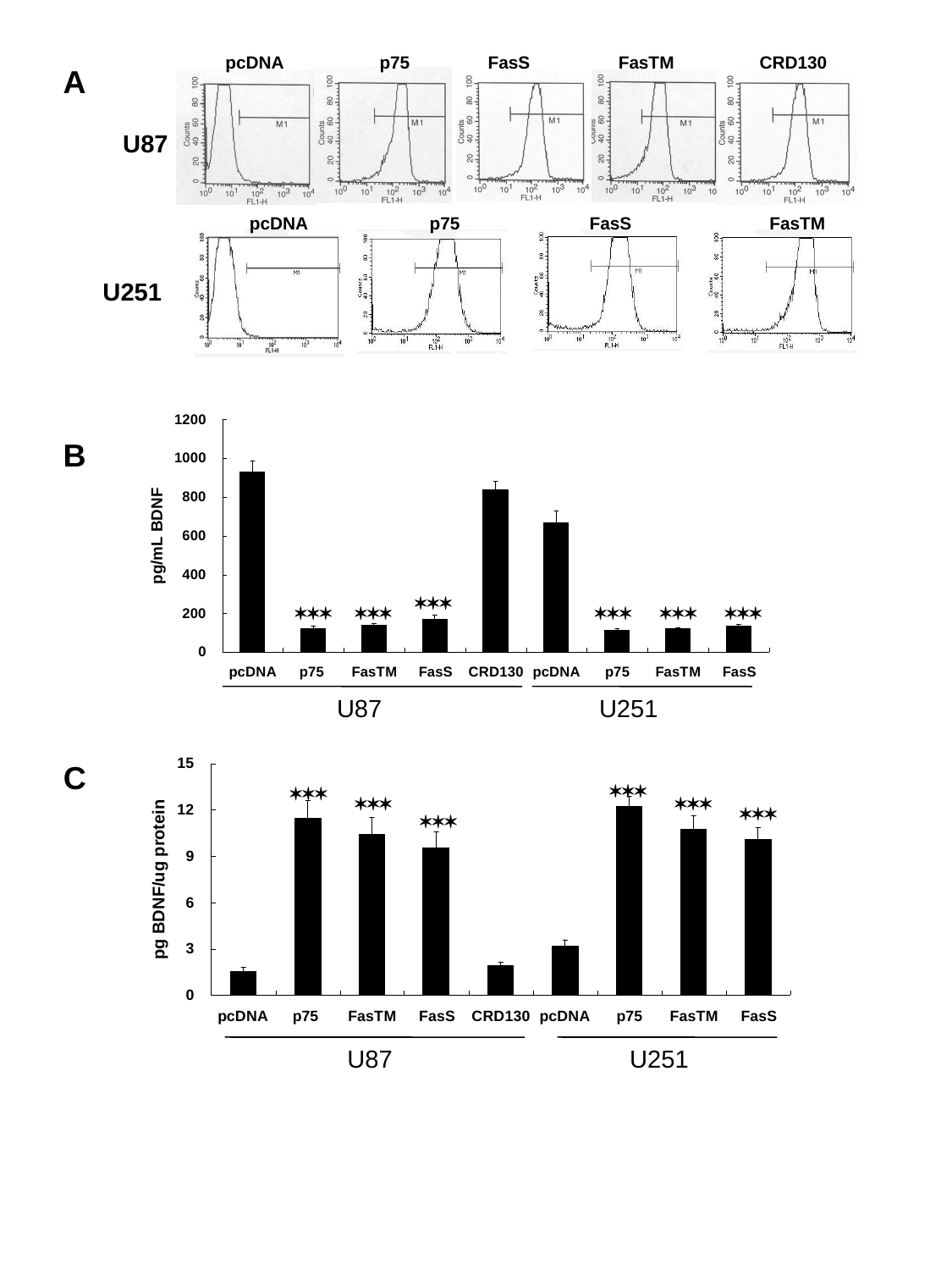

pcDNA
p75
FasS
FasTM
CRD130
A
U87
pcDNA
p75
FasS
FasTM
U251
U87
U251
B






C






U87
U251

Supplement: Figure S3 — (A) Expression and topography of the full-length, chimeric constructs (p75FasTM and p75FasS) and the neurotrophin-binding mutant p75CRD130 at the plasma membrane were confirmed by flow cytometric analysis using a p75NTR extracellular domain-specific antibody. U87pcDNA and U251pcDNA glioma cells were used as controls. (B–D) The p75NTR cleavage-resistant constructs retain their ability to bind ligand (C and D). Conditioned medium (B) and total cell lysates (C) from U87 and U251 cells expressing full-length p75, p75FasTM, p75FasS, and p75CRD130 were analyzed by ELISA for their ability to bind BDNF. The p75NTR-negative U87pcDNA and U251pcDNA were used for comparison. Expression of p75NTR full-length or the cleavage-resistant chimeric proteins (p75FasTM and p75FasS) produced a shift in BDNF localization from the conditioned medium to the cell lysate, consistent with the binding of BDNF to p75NTR. In contrast, cells expressing the ligand-binding mutant p75CRD130 did not produce a shift in BDNF localization in accordance with the in ability to bind ligand. Values shown are the mean ± standard error of the mean (s.e.m.) for a single experiment. Similar results were seen in three independent experiments; triple asterisks (∗∗∗) indicate p < 0.001 as compared to pcDNA control for each cell line (one-way ANOVA with the Neuman-Keuls post-test). (442 KB PPT) [file pbio.0060289.sg003.ppt]

## Slide 1
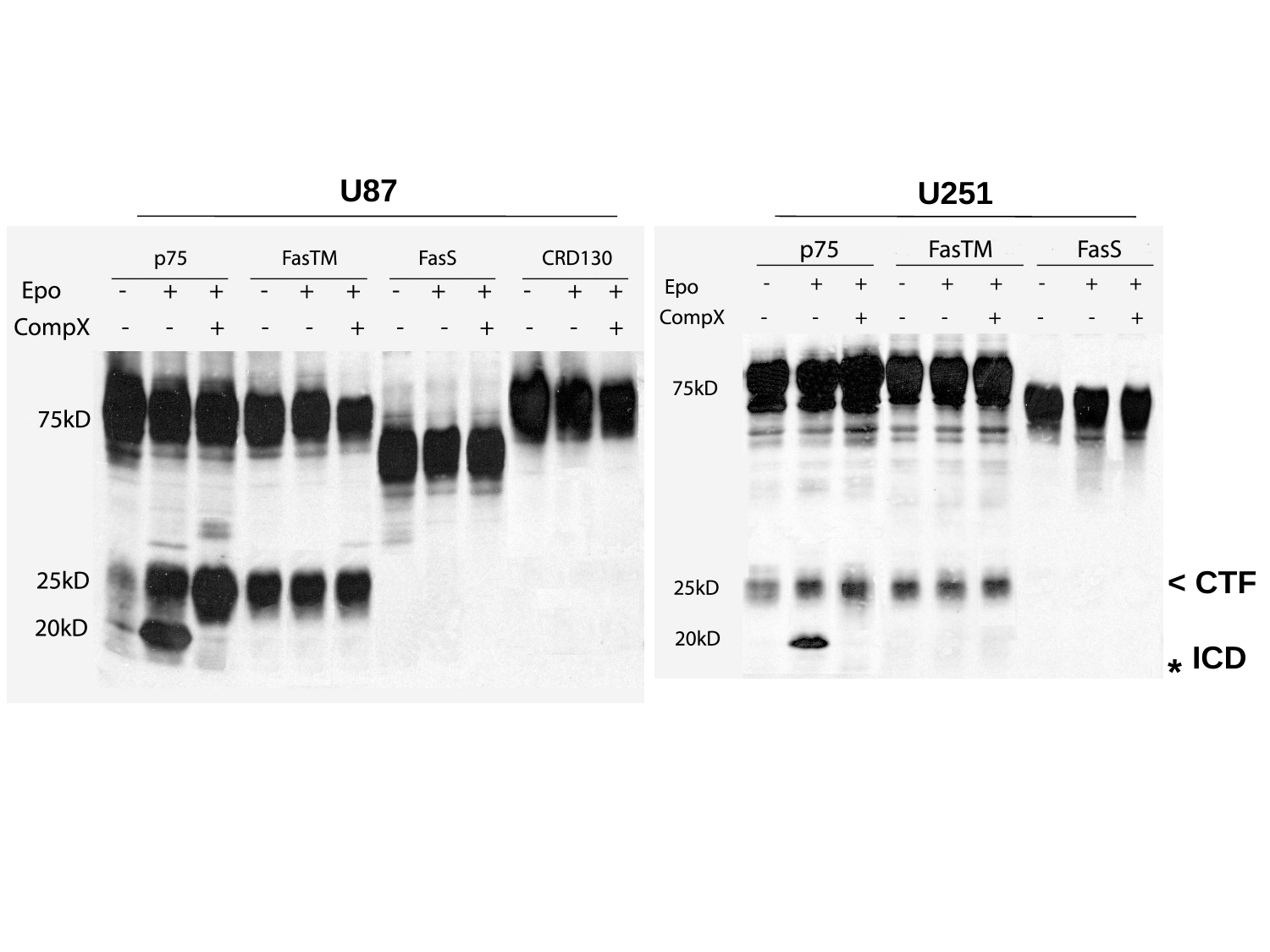

U87
U251
< CTF
* ICD

Supplement: Figure S4 — U87 (left panel) and U251 (right panel) stably transfected with p75NTR wild type, the cleavage-resistant chimeras (FasS and FasTM), or the neurotrophin-binding mutant CRD130 were treated with the proteasome inhibitor epoxomicin (Epo, 2 μM) and/or the specific inhibitor of γ-secretase, Compound X (CompX, 2 μM) for 4 h. Western blots for p75NTR were probed with an antibody specific to the cytoplasmic domain of p75NTR which detects full-length (75 kDa), CTF (indicated by the less than symbol [<]; 25 kDa), and ICD (indicated by the asterisk [*]; 19 kDa) peptides. In cell lysates from glioma cells expressing p75FasTM chimera, only the 24-kDa fragment was detected, whereas the p75FasS chimera and the p75CRD130 ligand binding site mutant did not display any cleaved products. (1.30 MB PPT) [file pbio.0060289.sg004.ppt]

## Slide 1
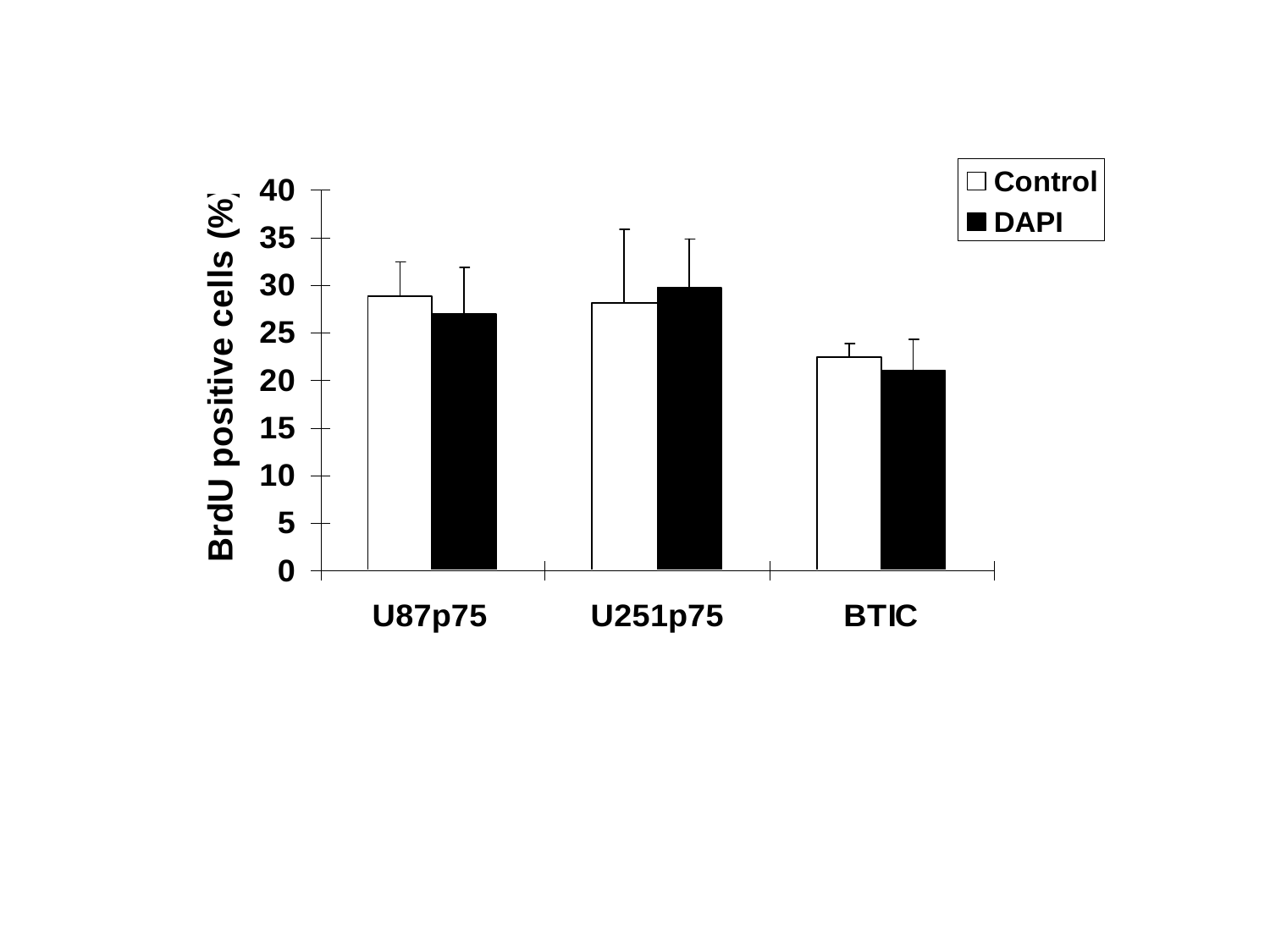

Supplement: Figure S5 — u87p75NTR- (A), U251p75NTR-, (B) or p75NTR-positive BTICs established from a patient GBM specimen (C) were implanted intracerebrally into SCID mice. Three days (U87p75NTR and U251p75NTR) or 5 d (BTIC) later, mice were administered s.c. 10 mg/kg γ-secretase inhibitor DAPT or vehicle (corn oil) alone, once/day for 2–3 wk (three to five mice/group). Bromodeoxyuridine (BrdU) was injected into the tumor-bearing mice 24 h prior to their sacrifice. Frozen brain sections were stained with an antibody against BrdU and counterstained with toluidine blue to visualize the cell nucleus. Cells that had divided during the 24 h prior to sacrifice stained positively for BrdU, and the percentage of BrdU-positive cells were counted. Bar graph represents the percentage of BrdU-positive cells in five consecutive fields. The γ-secretase inhibitor DAPT had no significant effect on proliferation in vivo of the p75NTR-glioma cells as compared to their untreated controls. (60 KB PPT) [file pbio.0060289.sg005.ppt]
